# *SLIT2* promoter hypermethylation predicts disease progression in chronic myeloid leukemia

**DOI:** 10.1186/s40001-022-00899-2

**Published:** 2022-11-21

**Authors:** De-long Wu, Yun Wang, Ting-juan Zhang, Ming-qiang Chu, Zi-jun Xu, Qian Yuan, Ji-chun Ma, Jiang Lin, Jun Qian, Jing-dong Zhou

**Affiliations:** 1 Department of Hematology, Affiliated People’s Hospital of Jiangsu University, 8 Dianli Rd., Zhenjiang, 212002 Jiangsu People’s Republic of China; 2 Zhenjiang Clinical Research Center of Hematology, Zhenjiang, Jiangsu People’s Republic of China; 3 The Key Lab of Precision Diagnosis and Treatment of Zhenjiang City, Zhenjiang, Jiangsu People’s Republic of China; 4 Laboratory Center, Affiliated People’s Hospital of Jiangsu University, 8 Dianli Rd., Zhenjiang, 212002 Jiangsu People’s Republic of China; 5 Department of Oncology, Affiliated People’s Hospital of Jiangsu University, Zhenjiang, Jiangsu People’s Republic of China; 6 Department of Oncology, Dongtai People’s Hospital, Dongtai, Jiangsu People’s Republic of China

**Keywords:** *SLIT2*, Methylation, Expression, Progression, Chronic myeloid leukemia

## Abstract

**Background:**

Aberrant DNA methylation plays a crucial role in the progression of myeloid neoplasms. Previously, our literature reported that slit guidance ligand 2 (*SLIT2*) promoter methylation was associated with disease progression and indicated a poor prognosis in patients with myelodysplastic syndrome. Herein, we further investigated the clinical implications and role of *SLIT2* promoter methylation in patients with chronic myeloid leukemia (CML).

**Methods:**

The level of *SLIT2* promoter methylation was determined in 104 CML patients, and its clinical significance was analyzed. Moreover, demethylation studies were performed in K562 cells to determine the epigenetic mechanism by which *SLIT2* promoter methylation is regulated in CML.

**Results:**

The level of *SLIT2* promoter methylation was similar between CML patients and controls. However, deeper analysis revealed that the *SLIT2* promoter methylation level in the accelerated phase (AP) and blast crisis (BC) was markedly higher than that in the chronic phase (CP) and controls. Additionally, a marked difference was identified between the *SLIT2* promoter hypermethylated and non-hypermethylated groups among CML patients grouped by clinical stage. The frequency of *SLIT2* hypermethylation was markedly increased with the progression of clinical stage, that is, it was the lowest in CP samples (12/80, 15%), higher in AP samples (4/8, 50%) and the highest in BC samples (11/16, 69%). Importantly, the level/density of *SLIT2* promoter methylation was significantly higher in the advanced stage than in the early stage among the 6 tested paired CML patients. Epigenetically, the expression of the *SLIT2*-embedded non-coding genes *SLIT2-IT1* and *miR-218* expression was decreased in patients with CML. *SLIT2* promoter hypermethylated cases had a markedly lower *SLIT2-IT1* expression level than *SLIT2* promoter non-hypermethylated cases. Moreover, *SLIT2-IT1* and *miR-218* expression was remarkably upregulated in a dose-dependent manner after demethylation treatment of K562 cells.

**Conclusions:**

Hypermethylation of the *SLIT2* promoter is correlated with disease progression in CML. Furthermore, *SLIT2* promoter methylation may function by regulating the expression of the *SLIT2*-embedded non-coding genes *SLIT2-IT1* and *miR-218* during CML progression.

**Supplementary Information:**

The online version contains supplementary material available at 10.1186/s40001-022-00899-2.

## Background

Chronic myeloid leukemia (CML) is initiated by the reciprocal translocation t(9;22)/Philadelphia (Ph) chromosome, which leads to the formation of the *BCR::ABL1* fusion protein with aberrant tyrosine kinase activity [1]. The treatment of CML is also based on the inhibition of aberrant tyrosine kinase activity as targeted therapy [1]. The typical clinical course of CML includes the initial stage of the chronic phase (CP) and the advanced/aggressive stage of the accelerated phase (AP) and blast crisis (BC) during disease progression [1]. Although CML is cytogenetically/genetically homogenous at the earlier stage, considerable genetic and/or epigenetic heterogeneity is identified in the later stage of CML [1, 2]. Cytogenetic and genetic abnormalities are pathogenetically associated with the progression of CML [2, 3]. Recently, aberrant DNA methylation, which plays a crucial role in the progression of CML, has attracted our attention [4, 5].

The slit guidance ligand (SLIT) family members (*SLIT1*/*SLIT2*/*SLIT3*) are highly conserved secreted glycoproteins that regulate various physiologic processes, such as neuronal axon guidance, cell proliferation, cell migration, and vascularization, by binding to roundabout (ROBO) receptors (*ROBO1*/*ROBO2*/*ROBO3*/*ROBO4*) [6]. The SLIT/ROBO signaling pathway was originally recognized in the nervous system and functions in neuronal axon guidance and is also considered an important regulator of multiple physiological and oncogenic processes [6, 7]. Recently, an increasing number of studies have reported the dysregulation of SLIT/ROBO signaling pathways in a variety of human cancers [7]. Epigenetic silencing of SLITs mediated by promoter hypermethylation plays a vital role in cancer initiation and progression [10]. Accordingly, a number of studies have shown that SLITs/ROBOs are frequently downregulated and have anticancer roles in the advanced stage of several solid tumors [8, 9]. However, several other studies have demonstrated an oncogenic role during cancer development [8, 9]. Interestingly, the SLIT2-embedded non-coding RNA (ncRNA) *miR-218* was found to be downregulated and to act as a tumor suppressor gene in human cancers in most studies [11]. In addition, the other SLIT2-embedded ncRNA *SLIT2-IT1* has rarely been investigated.

Previously, our study reported that hypermethylation of the *SLIT2* promoter was associated with disease progression in myelodysplastic syndrome (MDS) and predicted poor clinical outcome in both MDS and acute myeloid leukemia (AML) [12]. Moreover, *SLIT2* promoter methylation exerted its function by repressing the expression of two SLIT2-embedded ncRNAs, *SLIT2-IT1* and *miR-218* (*SLIT2-IT1*/*miR-218*), in MDS and AML [12]. However, the pattern and clinical implications of *SLIT2* promoter methylation in CML remain poorly defined. Herein, on the basis of previous research, we further determined the pattern, clinical implication and role of *SLIT2* promoter methylation in patients with CML.

## Materials and methods

### Subjects and samples

The current study included 104 de novo CML patients (80 in CP stage, 8 in AP stage and 16 in BC stage) and 51 healthy donors (age and sex-matched). The diagnosis and clinical stages of CML were established by clinical manifestation and laboratory examination of peripheral blood (PB)/bone marrow (BM), and were confirmed by molecular detection of the *BCR::ABL1* transcript. The *BCR::ABL* transcript detection was quantified using real-time quantitative PCR (RT-qPCR) established previously [13]. BM samples collected from the subjects were further used for the extraction of BM mononuclear cells (BMMNCs) using Lymphocyte Separation Medium (Solarbio, Beijing, China) by gradient centrifugation.

### Cell line, cell culture and demethylation treatment

The human CML cell line K562 was cultured in RPMI 1640 medium (Solarbio, Beijing, China) with 10% fetal calf serum (ExCell, Shanghai, China) and grown in a 5% CO_2_ humidified atmosphere at 37 °C. For demethylation treatment, K562 cells were treated with 5-aza-2’-deoxycytidine (5-aza-dC) (Sigma‒Aldrich, St. Louis, MO) at final concentrations of 0 μM, 1 μM, 2 μM, and 4 μM for 3 days. All treated cells were cultured until harvested for extraction of total RNA and DNA.

### RNA isolation, reverse transcription and RT-qPCR

Total RNA was isolated using TRIzol reagent (Invitrogen, Carlsbad, CA), followed by reverse transcription to synthesize cDNA for miRNA and long non-coding RNA (lncRNA) detection [12, 14]. RT-qPCR was performed to examine *SLIT2-IT1*/*miR-218* expression by AceQ qPCR SYBR Green Master Mix (Vazyme Biotech Co., Piscataway, NJ). The primers for *SLIT2-IT1*/*miR-218* expression were previously reported [12]. Relative *SLIT2-IT1*/*miR-218* transcript expression was calculated using the 2− ^∆∆CT^ formula according to the *ABL1* transcript.

### DNA isolation, chemical modification and RT-qMSP

The isolation and modification of genomic DNA was performed using Puregene Blood Core Kit B and EpiTect Bisulfite Kit (QIAGEN, Duesseldorf, Germany) as described previously [15, 16]. Real-time quantitative methylation-specific PCR (RT-qMSP) was first used to evaluate *SLIT2* promoter methylation with AceQ qPCR SYBR Green Master Mix (Vazyme Biotech Co., Piscataway, NJ). The primers for *SLIT2* promoter methylation detection were as reported [12]. Relative *SLIT2* promoter methylation was counted using the 2− ^∆∆CT^ formula as referred to *ALU* methylation.

### BSP

Bisulfite sequencing PCR (BSP) was further performed to detect *SLIT2* promoter methylation using TaKaRa Taq^™^ Hot Start Version (Tokyo, Japan). The primers for *SLIT2* promoter methylation detected by BSP were reported previously [17]. The details of BSP can be found in our previous study [17]. Six independent clones from each specimen were selected for Sanger sequencing (BGI, Shanghai, China).

### Statistics

Statistics were accomplished using SPSS 20.0 and GraphPad Prism 5.0 software packages. The differences in continuous variables between the two groups were compared by Mann‒Whitney’s U test. The differences in categorical variables between the two groups were compared by Pearson Chi-square analysis or Fisher’s exact test. The association of *SLIT2* promoter methylation with *SLIT2-IT1*/*miR-218* expression was analyzed by Spearman correlation test. Among all statistical analyses, a two-tailed *P* value < 0.05 was considered statistically significant.

## Results

### SLIT2 promoter methylation in CML patients

**Fig. 1:**
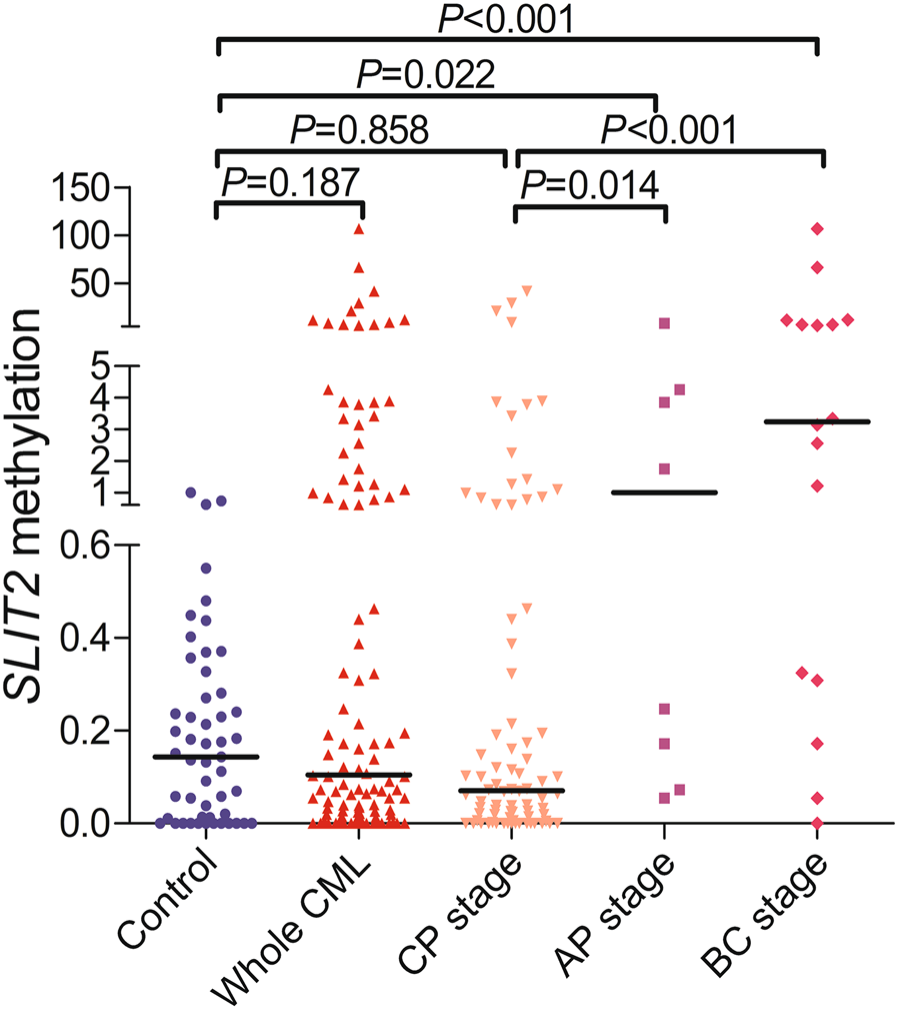
Relative *SLIT2* promoter methylation level in controls and CML patients. *SLIT2* promoter methylation was detected by RT-qMSP in controls and whole CML patients as well as different stages of CML patients (CP stage, AP sage, and BC stage) were presented with scatter plots. The median level of *SLIT2* promoter methylation in each group was shown with horizontal line

Previously, we reported the pattern of *SLIT2* promoter methylation in patients with MDS and AML and revealed that *SLIT2* promoter methylation was correlated with disease progression [12]. Herein, we further detected *SLIT2* promoter methylation in CML patients by RT-qMSP as previously described. The results showed that the *SLIT2* promoter methylation level was similar between CML patients and controls (*P* = 0.187, Fig. 1). However, further analysis revealed that the *SLIT2* promoter methylation level in the CML-AP and CML-BC stages was markedly higher than that in the CML-CP stage (*P* = 0.014 and < 0.001, respectively, Fig. 1) and in controls (*P* = 0.022 and < 0.001, respectively, Fig. 1). The above results indicated that *SLIT2* promoter methylation is correlated with an advanced stage of CML and may correlate with disease progression.

### Association between SLIT2 promoter methylation and clinicopathological characteristics of CML patients

To determine the correlation between *SLIT2* promoter methylation and clinicopathological characteristics of CML, the whole cohort of CML patients was divided into two groups based on the previously set cut-off points [12]. No statistical differences were found between the *SLIT2* promoter hypermethylated and non-hypermethylated groups with respect to sex, age, hemoglobin, karyotype and BCR-ABL transcript status (Table 1). However, *SLIT2* promoter hypermethylated cases exhibited lower white blood cells (WBCs) and platelets than *SLIT2* promoter non-hypermethylated cases (*P* < 0.001 and = 0.006, respectively, Table 1). Notably, a marked difference was identified between the *SLIT2* promoter hypermethylated and non-hypermethylated groups in CML patients grouped by clinical stage (*P* < 0.001, Table 1). The frequency of *SLIT2* hypermethylation was markedly increased with the progression of clinical stage, that is, it was the lowest in CML-CP samples (12/80, 15%), higher in CML-AP samples (4/8, 50%) and the highest in CML-BC samples (11/16, 69%) (*P* < 0.001, Table 1). These results further confirmed that *SLIT2* promoter methylation was correlated with an advanced stage of CML and may correlate with disease progression.

**Table 1:** Comparison of clinicopathological characteristics between *SLIT2* hypermethylated and non-hypermethylated CML patients

Patients’ parameters	*SLIT2* promoter non-hypermethylated (*n* = 77)	*SLIT2* promoter hypermethylated (*n* = 27)	*P* value
Sex, male/female	50/27	15/12	0.489
Median age, years (range)	51 (15–88)	46 (20–75)	0.680
Median WBC, × 10^9^/L (range)	128.4 (31.5–413.8)	41.6 (21.7–293.4)	< 0.001
Median hemoglobin, g/L (range)	101 (57–146)	92 (50–152)	0.277
Median platelet, × 10^9^/L (range)	387 (22–1489)	250 (16–914)	0.006
Karyotype			0.438
t(9;22)	53 (69%)	14 (52%)	
t(9;22) with additional alteration	10 (13%)	4 (15%)	
Normal karyotype	4 (5%)	3 (11%)	
No data	10 (13%)	6 (22%)	
Clinical stage			< 0.001
CP	68 (88%)	12 (44%)	
AP	4 (5%)	4 (15%)	
BC	5 (7%)	11 (41%)	
*BCR::ABL1* transcript (relative copy)	210 (16.9–3784.8)	239.1 (13.8–14464.7)	0.366

*WBC* white blood cell, *CP* chronic phase, *AP* accelerated phase, *BC* blast crisis

### SLIT2 promoter methylation alteration during disease progression in paired CML patients

**Fig. 2:**
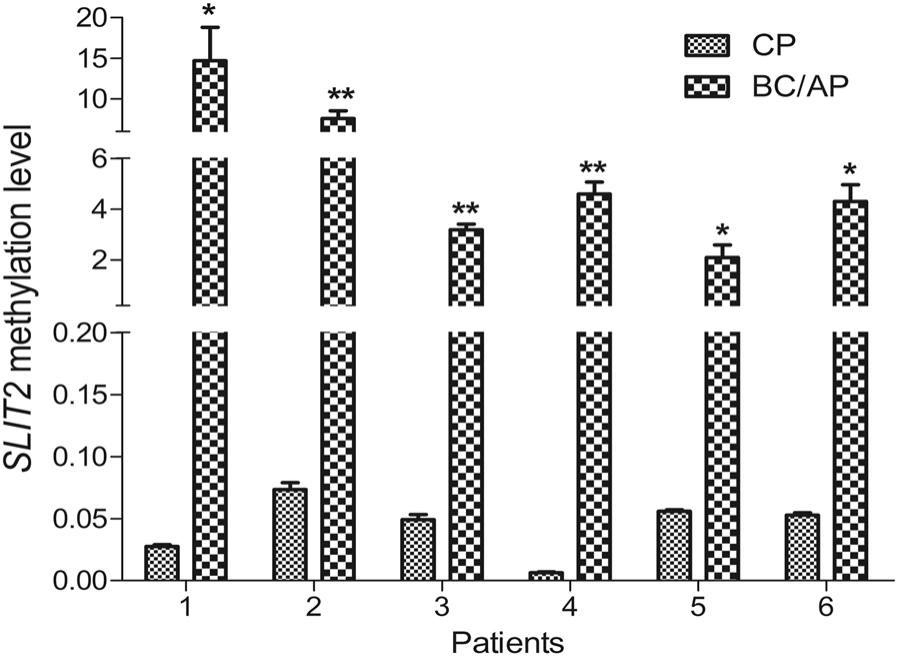
Relative *SLIT2* promoter methylation level changes during disease progression in six paired CML patients. *SLIT2* promoter methylation was detected by RT-qMSP in paired CML patients before and after disease progression. *P < 0.05; **P < 0.01; ***P < 0.001

**Fig. 3:**
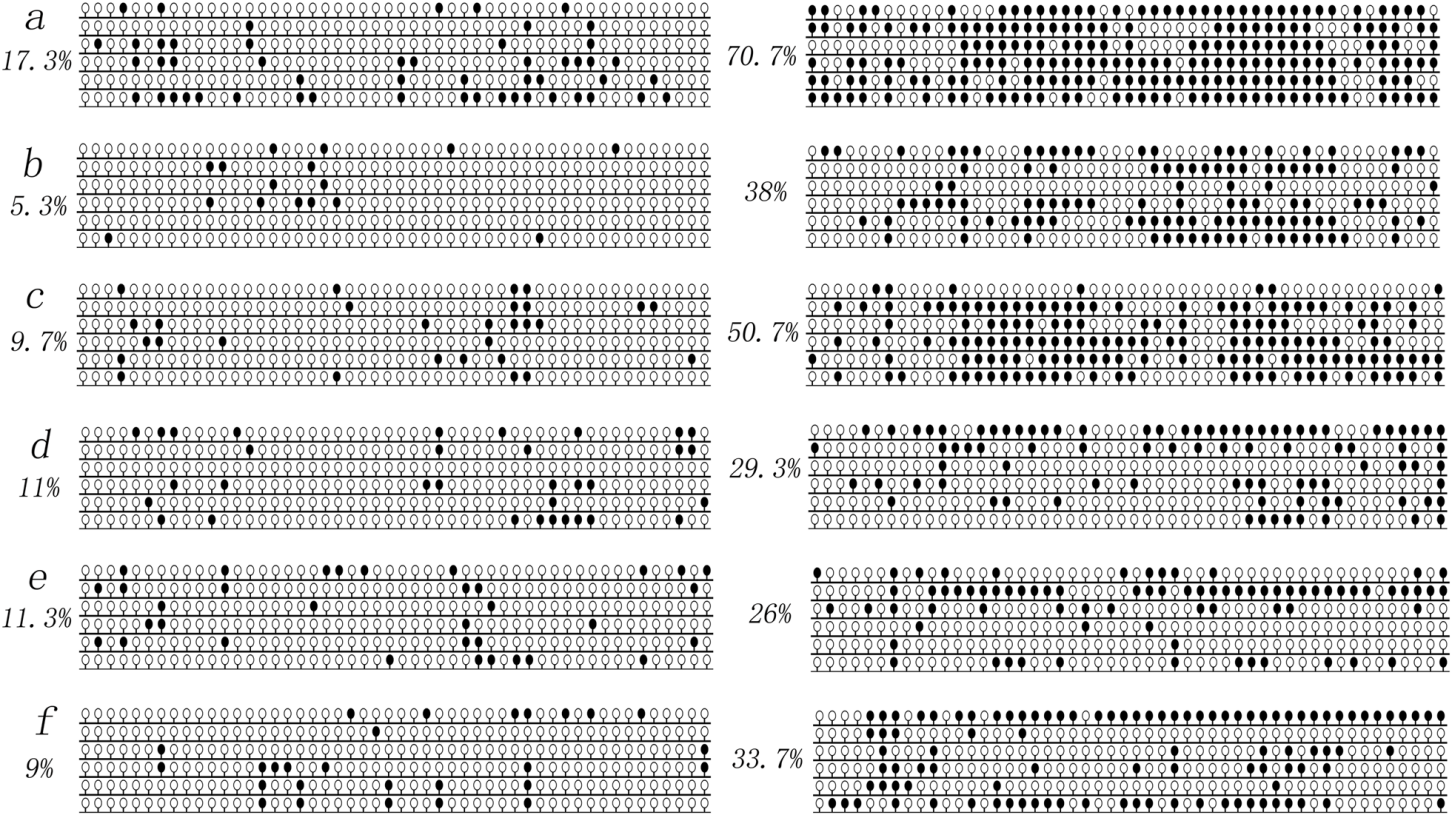
*SLIT2* promoter methylation density alterations during disease progression in six paired CML patients. White cycle: unmethylated CpG dinucleotide; Black cycle: methylated CpG dinucleotide. *SLIT2* promoter methylation density in CP stage in Patient a-f were 17.3%, 5.3%, 9.7%, 11%, 11.3% and 9%, whereas in AP/BC stage in Patient a-f were 70.7%, 38%, 50.7%, 29.3%, 26% and 33.7% (*P* = 0.004, Paired *T* test)

Given the results above, we hypothesized that *SLIT2* promoter methylation was correlated with disease progression in CML. To test this hypothesis, we further examined *SLIT2* promoter methylation in paired CML patients during disease progression. By RT-qMSP, the level of *SLIT2* promoter methylation was significantly upregulated in the advanced stage compared with the early stage among the tested 6 paired CML patients (Fig. 2). Moreover, the *SLIT2* promoter methylation density in these paired patients was further detected by BSP (Fig. 3) and was closely correlated with the results detected by RT-qMSP (*R* = 0.895, *P* < 0.001, Additional file 1: Fig S1). Taken together, these results suggest that *SLIT2* promoter methylation is correlated with disease progression in CML.

### Epigenetic regulatory effects of SLIT2 promoter methylation in CML

**Fig. 4:**
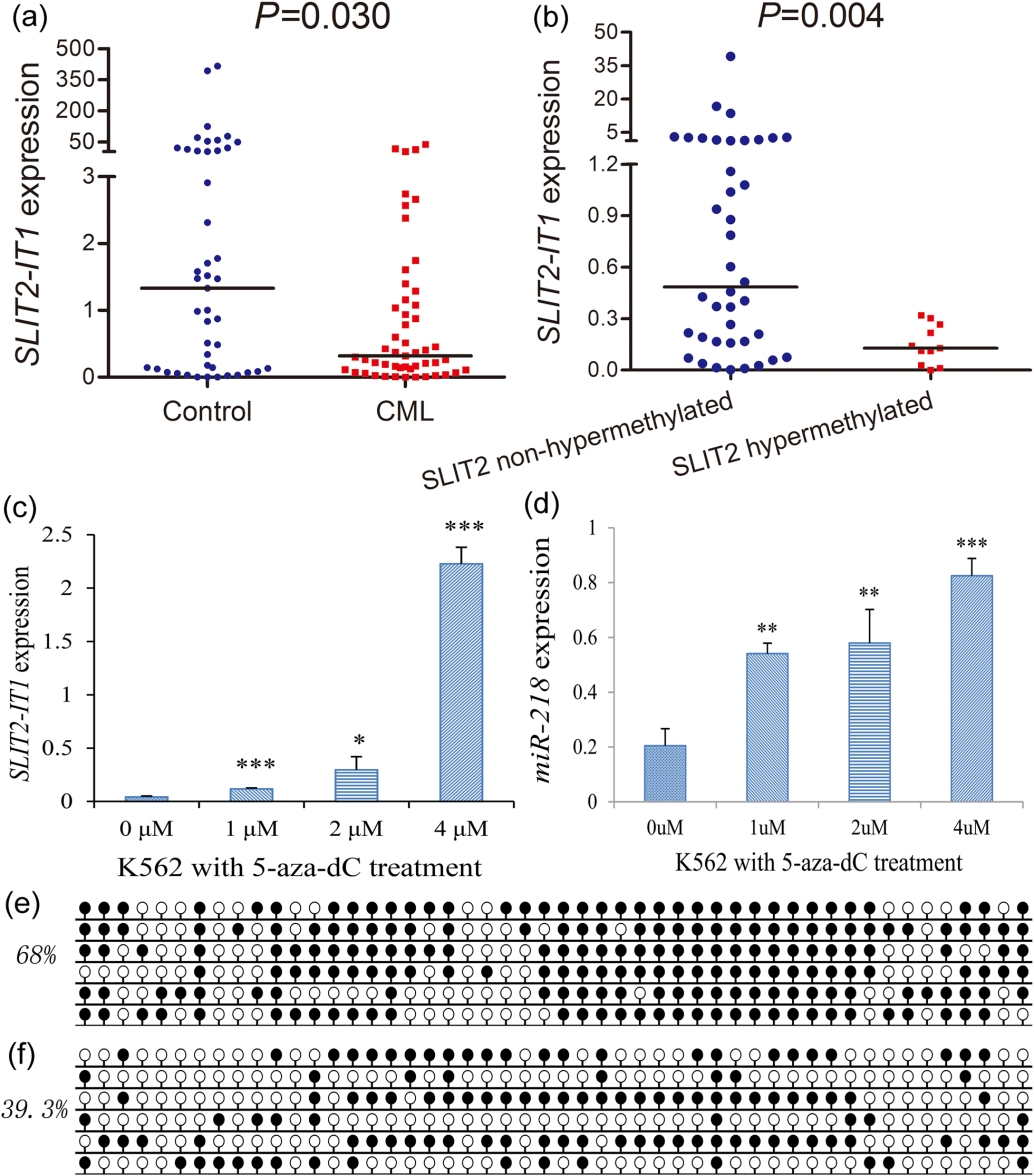
Transcriptional regulatory effects of *SLIT2* promoter methylation on *SLIT2-IT1*/*miR-218* expression in CML. **a** Relative *SLIT2-IT1* expression level in CML patients; **b** relative *SLIT2-IT1* expression between *SLIT2* promoter non-hypermethylated and hypermethylated groups; **c** *SLIT2-IT1* expression before and after 5-aza-dC treatment with different dose; **d** *miR-218* expression before and after 5-aza-dC treatment with different dose; **e** *SLIT2* promoter methylation density before 5-aza-dC treatment; **f** *SLIT2* promoter methylation density after 5-aza-dC treatment (4 μM)

Previously, we revealed that *SLIT2* promoter methylation was associated with *SLIT2*-embedded ncRNAs *SLIT2-IT1*/*miR-218* expression but not *SLIT2* expression in MDS and AML. Herein, we further detected *SLIT2-IT1*/*miR-218* expression in 51 CML patients with available mRNA samples matched to DNA samples. *SLIT2-IT1* expression was markedly decreased (*P* = 0.030, Fig. 4a), whereas *miR-218* expression was nearly undetectable in CML patients. Moreover, although *SLIT2-IT1* expression exhibited a weak negative association with *SLIT2* promoter methylation in CML patients (R = − 0.289, *P* = 0.039, n = 51), cases with *SLIT2* promoter hypermethylation had a markedly lower *SLIT2-IT1* expression level than those without *SLIT2* promoter hypermethylation (*P* = 0.004, Fig. 4b). To further verify the epigenetic regulatory effects of *SLIT2* promoter methylation on the ncRNAs *SLIT2-IT1*/*miR-218*, we performed demethylation treatment of the CML cell line K562 with 5-aza-dC. With the decreased density of *SLIT2* promoter methylation, *SLIT2-IT1*/*miR-218* expression was markedly upregulated in a dose-dependent manner after 5-aza-dC treatment (Fig. 4c–f). Collectively, these results support the epigenetic regulatory effects of *SLIT2* promoter methylation on the expression of SLIT2-embedded ncRNAs *SLIT2-IT1*/*miR-218* in CML.

## Discussion

The SLIT/ROBO signaling pathway has been implicated in the regulation of developmental processes and physiological processes [6, 7]. SLIT/ROBO signaling plays crucial roles in a number of cell signaling pathways including axon guidance, angiogenesis, cell proliferation, cell apoptosis and cell motility [6, 7]. Moreover, inactivation of SLITs/ROBOs expression mediated by promoter methylation in cells can lead to cancer initiation and progression [10]. Notably, several studies have demonstrated that SLITs/ROBOs are frequently downregulated in the advanced stage of various solid tumors [8, 9]. This evidence indicated that the SLIT/ROBO signaling pathway may play a crucial role in cancer progression than cancer initiation. Previously, we reported that *SLIT2* promoter methylation through the inactivation of *SLIT2-IT1*/*miR-218* expression may play a key role in MDS progression by affecting cell proliferation, apoptosis and colony formation both in vitro and in vivo [12]. In the current study, we observed that *SLIT2* promoter hypermethylation was associated with lower WBCs and platelet in CML, which suggests that *SLIT2* promoter hypermethylation may be associated with hematopoietic stem cells differentiation fate. Accordingly, further functional studies are needed to determine the direct role of aberrant *SLIT2* promoter methylation in leukemogenesis during CML progression.

To date, the mechanisms involved in CML progression have been preliminarily identified. Cytogenetic aberrations, such as double t(9;22)/Ph chromosome, trisomy chromosome 8, i(17q), trisomy chromosome 19, t(3;21) and t(7;11), and molecular alterations, including *TP53* mutations, *RAS* mutations and increased *BCR::ABL1* transcript levels, are pathogenetically correlated with the progression of CML [2, 3]. Moreover, epigenetic alterations, such as aberrant DNA methylation, have also been identified to play a vital role in the disease evolution of CML [4, 5]. For instance, Li et al. revealed that *SHP-1* hypermethylation was involved in CML evolution through the regulation of the *BCR::ABL1*, AKT, MAPK, MYC and JAK2/STAT5 signaling pathways [18]. Additionally, our research group has also revealed the correlation of *SOX30*, *ID4* and *DLX4* hypermethylation with disease progression in CML [19–21]. A recent study demonstrated that promoter hydroxymethylation of tumor suppressor genes *DAPK1*, *RIZ1*, *P16INK4A*, *RASSF1A* and *p14ARF*^*ARF*^ was a characteristic feature of CML disease progression and indicated poor imatinib response and poor overall survival of CML patients to imatinib therapy [22]. On the basis of our previous study [12], we further investigated *SLIT2* promoter methylation in another myeloid malignancy CML. In accordance with the results in MDS [12], *SLIT2* promoter methylation was also correlated with advanced clinical stage of CML, and played a crucial role in disease progression. Interestingly, Heller et al. observed up to 897 genes that were methylated at the time of progression but not at the time of diagnosis in CP-CML patients who progressed to AP/BC-CML using next-generation sequencing [4]. However, *SLIT2* promoter hypermethylation was not identified in this study [4], which may be attributed to differences in ethical considerations. Since this is the first report of *SLIT2* promoter hypermethylation in CML progression, prospective investigations are needed to confirm and expand our results.

DNA hypermethylation mainly functions by inactivating gene expression in cancer development. Although a few investigations have demonstrated the association between *SLIT2* promoter methylation and *SLIT2* expression in some types of solid tumors [23], our recent study revealed that *SLIT2* promoter methylation was correlated with the expression of SLIT2-embedded ncRNAs *SLIT2-IT1*/*miR-218* but not with *SLIT2* in AML [12]. Herein, we also explored the expression of *SLIT2-IT1* and *miR-218* expression in CML. The results showed that *SLIT2-IT1*/*miR-218* was significantly decreased in CML patients, and was negatively correlated with *SLIT2* promoter methylation. Moreover, demethylation studies also confirmed the epigenetic mechanism of *SLIT2* promoter methylation in regulating ncRNAs *SLIT2-IT1*/*miR-218* expression in CML. Taken together, these results indicated that *SLIT2* promoter hypermethylation may function by repressing *SLIT2-IT1*/*miR-218* expression during CML progression.

## Conclusion

Hypermethylation of the *SLIT2* promoter is correlated with disease progression in CML. Furthermore, *SLIT2* promoter methylation may regulate the expression of *SLIT2*-embedded non-coding genes *SLIT2-IT1*/*miR-218* during CML progression.

## Supplementary Information

**Additional file 1: Figure S1.** Correlation between SLIT2 methylation density detected by BSP and SLIT2 methylation level detected by RT-qMSP.

## Data Availability

The datasets used and/or analyzed during the current study are available from the corresponding author on reasonable request.

## Abbreviations

CML: Chronic myeloid leukemia
CP: Chronic phase
AP: Accelerated phase
BC: Blast crisis
Ph: Philadelphia
MDS: Myelodysplastic syndrome
AML: Acute myeloid leukemia
SLIT: Slit guidance ligand
ROBO: Roundabout
ncRNAs: Non-coding RNAs
PB: Peripheral blood
BM: Bone marrow
BMMNCs: BM mononuclear cells
5-aza-dC: 5-Aza-2’-deoxycytidine
lncRNA: Long non-coding RNA
RT-qPCR: Real-time quantitative PCR
RT-qMSP: Real-time quantitative methylation-specific PCR
BSP: Bisulfite sequencing PCR
WBC: White blood cell
