# Supplementary material for: SLIT2 promoter hypermethylation predicts disease progression in chronic myeloid leukemia
Source: Eur J Med Res. 2022 Nov 21;27:259. doi: 10.1186/s40001-022-00899-2 (PMC9677675; doi:10.1186/s40001-022-00899-2)
Supplement: Supplementary file 1 — Additional file 1: Figure S1. Correlation between SLIT2 methylation density detected by BSP and SLIT2 methylation level detected by RT-qMSP. [file 40001_2022_899_MOESM1_ESM.docx]

**Additional Figure Legends**

**
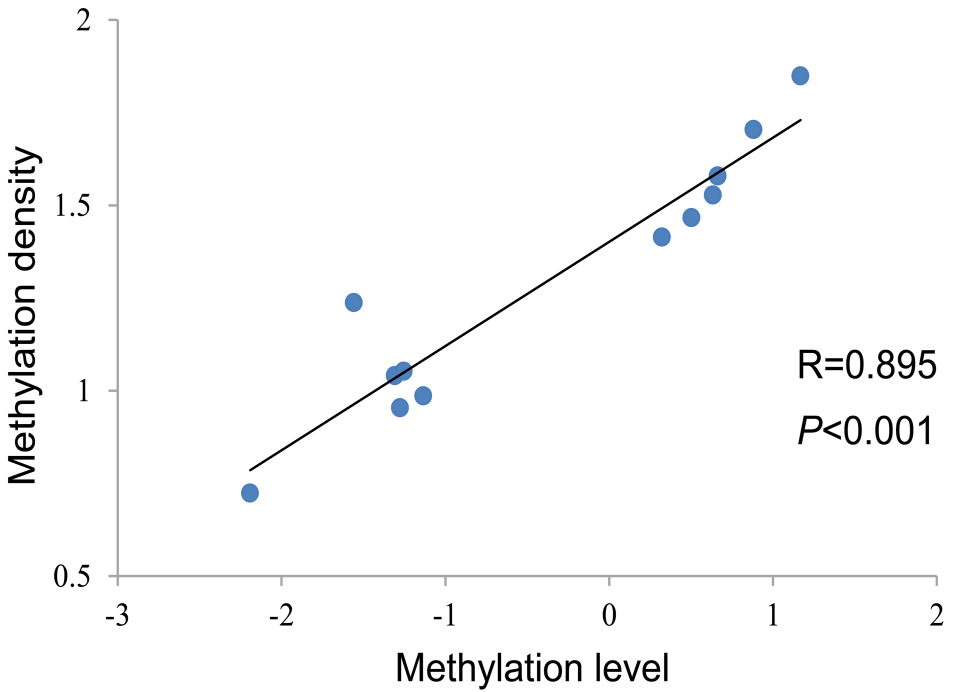
**

**Figure S1. Correlation between SLIT2 methylation density detected by BSP and SLIT2 methylation level detected by RT-qMSP.**
